# Export of recombinant proteins in *Escherichia coli *using ABC transporter with an attached lipase ABC transporter recognition domain (LARD)

**DOI:** 10.1186/1475-2859-8-11

**Published:** 2009-01-29

**Authors:** Chan Woo Chung, Jinsun You, Kyeongyeon Kim, Yuseok Moon, Hoeon Kim, Jung Hoon Ahn

**Affiliations:** 1 Korea Science Academy, #899, Tanggam 3-Dong, Busanjin-Gu, Busan, 614-822, Korea; 2 Department of Microbiology and Immunology, Medical Research Institute, Pusan National University School of Medicine, Busan, 602-739, Korea; 3 Biotherapeutic Division, GenExel-Sein Inc, Daejon, 305-701, Korea; 4 Institute for Gifted Students, Korea Advanced Institute of Science and Technology, Kusong-Dong 373-1, Yusong-Gu, Daejon 305-701, Korea

## Abstract

**Background:**

ATP binding cassette (ABC) transporter secretes the protein through inner and outer membranes simultaneously in gram negative bacteria. Thermostable lipase (TliA) of *Pseudomonas fluorescens *SIK W1 is secreted through the ABC transporter. TliA has four glycine-rich repeats (GGXGXD) in its C-terminus, which appear in many ABC transporter-secreted proteins. From a homology model of TliA derived from the structure of *P. aeruginosa *alkaline protease (AprA), lipase ABC transporter domains (LARDs) were designed for the secretion of fusion proteins.

**Results:**

The LARDs included four glycine-rich repeats comprising a β-roll structure, and were added to the C-terminus of test proteins. Either Pro-Gly linker or Factor Xa site was added between fusion proteins and LARDs. We attached different length of LARDs such as LARD0, LARD1 or whole TliA (the longest LARD) to three types of proteins; green fluorescent protein (GFP), epidermal growth factor (EGF) and cytoplasmic transduction peptide (CTP). These fusion proteins were expressed in *Escherichia coli *together with ABC transporter of either *P. fluorescens *or *Erwinia chrysanthemi*. Export of fusion proteins with the whole TliA through the ABC transporter was evident on the basis of lipase enzymatic activity. Upon supplementation of *E. coli *with ABC transporter, GFP-LARDs and EGF-LARDs were excreted into the culture supernatant.

**Conclusion:**

The LARDs or whole TliA were attached to C-termini of model proteins and enabled the export of the model proteins such as GFP and EGF in *E. coli *supplemented with ABC transporter. These results open the possibility for the extracellular production of recombinant proteins in *Pseudomonas *using LARDs or TliA as a C-terminal signal sequence.

## Background

Type I secretion system (T1SS) works in a continuous secretion process across both the inner and the outer membrane of Gram-negative bacteria [1,2]. The proteins involved in Type I secretion form a channel that exports proteins from the cytoplasm to the extracellular environment. An ATP binding cassette (ABC) protein recognizes the C-terminal signal sequence of the target protein, which is not cleaved during secretion and which hydrolyzes ATP for protein translocation [3,4]. Hence T1SSs are also known as ABC transporters. Membrane fusion protein (MFP) is exposed mainly to the periplasm and has one transmembrane segment anchored in the inner membrane [5]. MFP connects the ABC protein and outer membrane protein (OMP) during formation of the transport complex [6,7]. OMP is an outer membrane porin protein that forms a tunnel across the periplasm and the outer membrane [8]. The secreted protein contains a C-terminal targeting signal containing several repeats of the consensus sequence GGXGXD [9,10] and an extreme C-terminus motif [11].

In *Pseudomonas fluorescens*, the thermostable lipase (TliA) and protease genes are located upstream and downstream of ABC transporter operon [12]. Previously, the ABC transporter gene tliDEF was cloned and found to secrete the lipase in *E. coli *with concomitant expression of the lipase gene tliA [12]. The tliDEF was different in gene organization and amino acid sequence homology (~50%) with aprDEF of *P. aeruginosa*. Homologous expression of tliDEF and tliA in *P. fluorescens *by plasmid-mediated supplementation of these genes enhances the lipase content over 1000 times than the original *P. fluorescens *and 100 times more than *E. coli *harboring tliDEFA [13].

We have been curious about whether the C-terminal targeting signal sequence enables the secretion of other proteins. To assess this, we individually constructed some recombinant proteins including, green fluorescence protein (GFP; 238 amino acids), human epidermal growth factor (EGF; 53 aa), and cytoplasmic transduction peptide (CTP; 11 aa) as a fusion protein with lipase or the C-terminus of lipase. GFP was used as a model protein because it has a stable can-like shape consisting of a β-barrel [14,15] and has been used to visualize fusion proteins [7,15-17]. EGF is a growth factor that plays a role in the regulation of cell proliferation and differentiation [18]. CTP is a recently designed short peptide that is derived from protein transduction domain (PTD) of the human immunodeficiency virus [19,20], and which is used for the delivery of fused proteins into the cytoplasm of eukaryotic cells [21]. We assessed whether the aforementioned recombinant proteins were secreted in *E. coli *to elucidate the validity of the lipase C-terminal signal sequence, namely the lipase ABC transporter domain (LARD).

In the present investigation, we used two different ABC transporters, PrtDEF of *Erwinia chrysanthemi *and TliDEF of *P. fluorescens*. Since *E. chrysanthemi *is closely related to *E. coli*, we selected it as an efficient transporter for several recombinant proteins expressed in *E. coli*. We designed two different types of lipase C-terminal region, LARD0 and LARD1, in addition to the whole lipase (the biggest LARD). We constructed several combinations of recombinant proteins by fusing model proteins including GFP, EGF and CTP to translocation motifs including lipase itself, LARD0 or LARD1. These recombinant proteins were expressed with ABC transporters and checked for their secreting ability.

## Results

### LARD design

**Figure 1:**
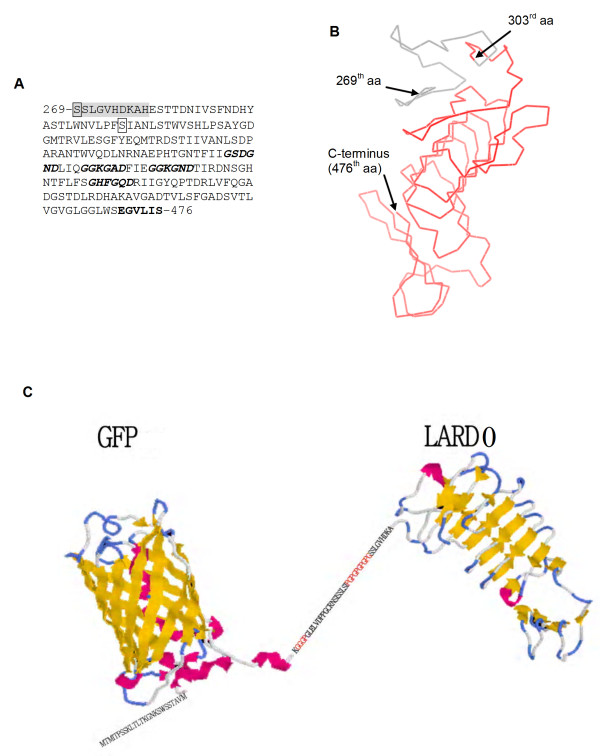
**Prospective structure of LARDs**. A. TliA C-terminal sequence. The first amino acids of LARD0 and LARD1 are indicated by boxes, a prospective hinge region is shaded, four glycine rich consensus sequences are italicized and in bold, and the extreme C-terminus motif is in bold. B. Schematic structural model of LARD. The probable structure of LARDs was determined using a homology modeling program. Two different LARDs were designed from the homology structure, making LARD0 (residues 269–476) and LARD1 (residues 303–476). C. Fusion protein of GFP and LARD0. A poly(Pro-Gly) linker and additional amino acids links between structural model of GFP and homology model of LARD0. Additional amino acids of N-terminus are added from the sequence of the plasmid.

The thermostable lipase (TliA) of *P. fluorescens *SIK W1 is comprised of 476 amino acids and has the characteristic C-terminal signal sequence recognized by the ABC transporter [12]. There are two distinctive features in the TliA C-terminal signal region: a glycine-rich consensus sequence, GGXGXD which is repeated four times, and an extreme C-terminus motif, EGVLIS, which consists of several hydrophobic residues preceded by an acidic residue, Glu [22]. The C-terminal signal sequence was designed as a tag for the fusion protein to be exported by ABC transporter. TliA could be divided into two different domains, N-catalytic domain and putative C-secretion/chaperon domain. The structural organization of TliA was assumed from the structures of three proteases in *P. aeruginosa *[23], *Serratia marcescens *[24] and *E. chrysanthemi *[25]. Although N-catalytic domain of TliA has no sequence homology with these proteases, the C-secretion/chaperon domain displays homology with the C-terminal region of these proteases. A comparative structural modeling was done, based on *P. aeruginosa *AprA, which has the highest homology with TliA at its C-secretion/chaperon domain. From structural modeling and sequence alignment of TliA and AprA, we positioned hinge region (residues 269–278 of TliA) bridging activity domain (residues 1–268) and secretion/chaperon domain (residues 279–476) (Figure 1A). Two different types of LARDs were designed to have the characteristic β-roll and additional loop region (Figure 1B). LARD0 was designed to incorporate residues 269–476 of TliA and was fused with the C-terminus of GFP (Figure 1C). A repetitive Pro-Gly linker was added to separate the domain of the fusion proteins from LARD0, which allows the fusion proteins to fold independently [26-28]. In case of LARD1, linkers were engineered to have a Factor Xa cleavage site. LARD1 also contained residues 303–476 of TliA.

### Identification of GFP-fusion protein

**Figure 2:**
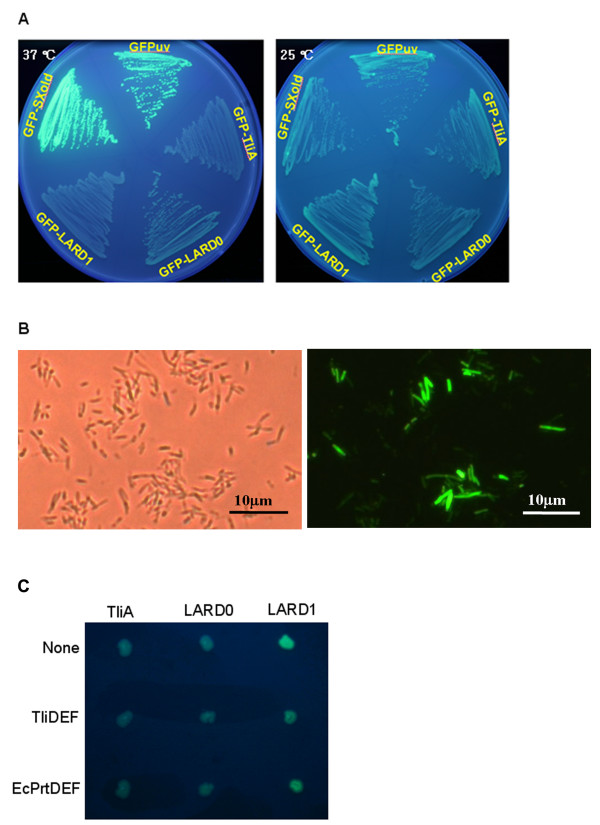
**Green fluorescence of GFP-fusion proteins**. A. Green fluorescence of GFP-fusion proteins without ABC transporter. *E. coli *harboring pGFPuv, pGFP-TliA, pGFP-LARD0, pGFP-LARD1 and pGFP-SXold (intermediate plasmid making the fusion proteins of GFP and a few additional amino acids) was streaked on LB containing ampicillin and grown at 37°C or 25°C. B. Green fluorescence under fluorescence microscope. *E. coli *harboring pGFP-LARD0 was viewed with a fluorescence microscope and photographs were obtained under light and excitation filter conditions with 1,000 magnification. C. Green fluorescence of GFP-fusion proteins with ABC transporters. *E. coli *harboring the GFP-fusion plasmids pGFP-TliA (TliA), pGFP-LARD0 (LARD0), or pGFP-LARD1 (LARD1) was viewed under UV illumination with or without ABC transporters. pACYC-184, pABC-ACYC, and pEcPrtDEF-184 lacked transporter genes, or had the genes of TliDEF or EcPrtDEF as the ABC transporter, respectively.

Before the secretion of GFP fusion protein was analyzed, we needed to check the expression of GFP-fusion proteins such as GFP-LARD0, GFP-LARD1, and GFP-TliA (GFP fused to entire TliA). These proteins were expressed in *E. coli *and their expected sizes were confirmed by Western blotting (data not shown). In addition, the fluorescence of GFP was demonstrated. Representative colonies of *E. coli *expressing these proteins were viewed under ultraviolet (UV) light (Figure 2A). The fluorescence of control GFP was strong in *E. coli *grown at 37°C, whereas fluorescences of GFP-LARD0, GFP-LARD1 and GFP-TliA were comparatively somewhat weak in *E. coli *grown at the same temperature but more evident in *E. coli *grown at 25°C. *E. coli *populations harboring the fusion proteins were observed under fluorescent microscope. All GFP-fusion proteins were evident upon fluorescence microscopy. However, not every *E. coli *cells in the population showed green fluorescence, as exemplified by cells harboring GFP-LARD1 fusion, and the intensity of fluorescence was variable (Figure 2B). Therefore, the fluorescence apparent to the unaided eye represented the total of the different fluorescence intensities. We anticipated that fluorescence would be evident around *E. coli *colonies if bacteria were able to secrete the GFP-fusion proteins via the ABC transporter. To assess this, individual *E. coli *colonies, with or without an ABC transporter, were UV-irradiated to identify the expression of GFP-fusion proteins (Figure 2C). *E. coli *harboring pGFP-TliA, pGFP-LARD0, or pGFP-LARD1 radiated visible fluorescence under UV irradiation in the presence of TliDEF or PrtDEF transporter, and in the absence of transporter. However, contrary to our expectation, fluorescence surrounding *E. coli *ABC transporter positive colonies expressing GFP fusion proteins was not evident. All *E. coli *colonies displayed green fluorescence after a prolonged storage (about a week) at refrigerator temperature. The presence of the ABC transporter did not affect the activation of GFP in GFP-fusion proteins. *E. coli *harboring pGFP-LARD1 showed a more intense fluorescence than *E. coli *harboring pGFP-TliA or pGFP-LARD0.

### Fusion of whole TliA to make fusion proteins

**Figure 3:**
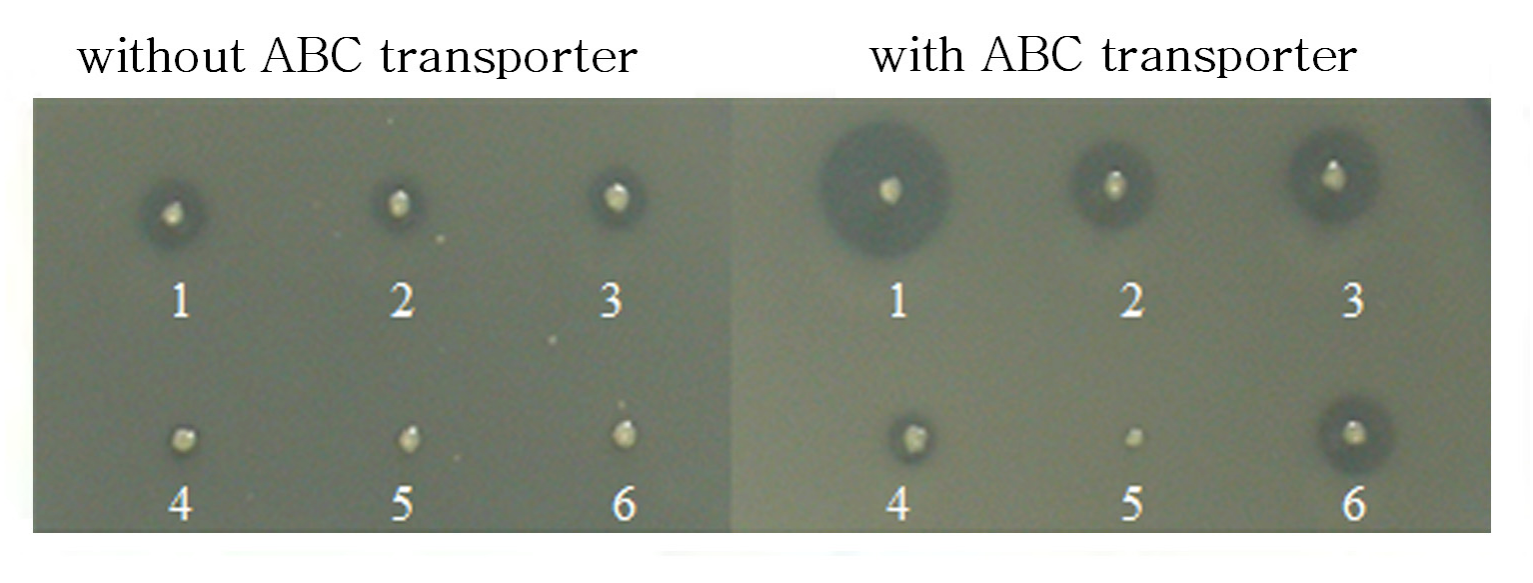
**Lipase activity of transformants harboring tliA-fusion gene**. Semi-quantitative estimation of the extracellular secretion of target protein containing lipase (TliA) was performed by comparing the sizes of lipolytic clear halos. *E. coli *transformants harboring tliA-fusion gene were prepared in two sets; with *P. fluorescens *ABC transporter (pABC-ACYC) and without ABC transporter (pACYC-184). After *E. coli *cells were incubated on the LAT plate at 25°C for 48 h, halos were observed; 1, *E. coli *(pTliA); 2, *E. coli *(pGFP-TliA); 3, *E. coli *(pEGF-TliA); 4, *E. coli *(pCTP-TliA); 5, *E. coli *(pTliA-CTP); 6, *E. coli *(pCTP-GFP-TliA).

**Figure 4:**
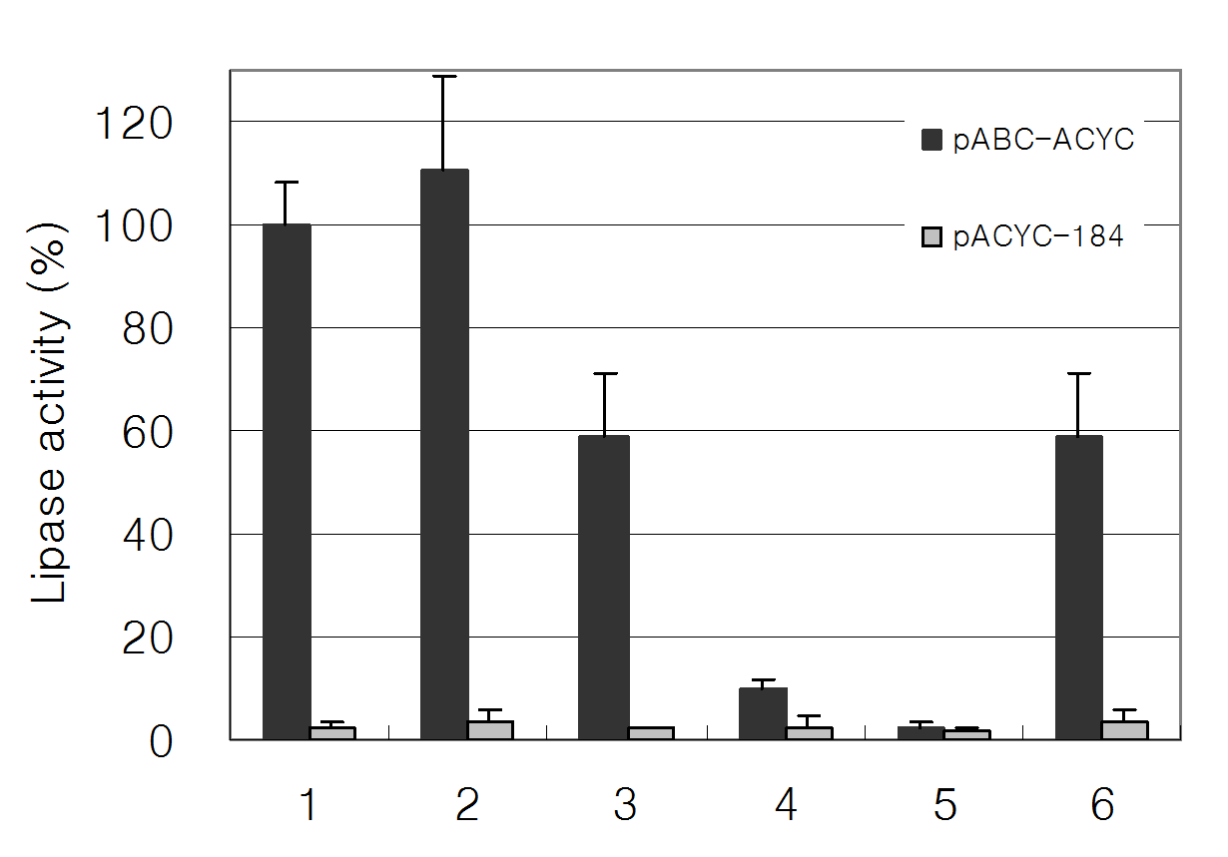
**Lipase activity of TliA-fusion protein secreted by *E. coli***. The lipase activities were represented as percentages of the activity secreted by *E. coli *harboring pTliA and pABC-ACYC. Each set of graph bar contains lipase activities with *P. fluorescens *ABC transporter (pABC-ACYC) or without ABC transporter (pACYC-184). Column 1, pTliA; Column 2, pGFP-TliA; Column 3, pEGF-TliA; Column 4, pCTP-TliA; Column 5, pTliA-CTP; Column 6, pCTP-GFP-TliA.

Independent of LARDs, we fused lipase (TliA) itself to recombinant proteins such as GFP, EGF, and CTP to check whether whole TliA could enable secretion of the recombinant proteins. The secretory phenotype could be traced via lipase activity. *E. coli *was cultivated on tributylin agar to detect the secretion of TliA-fusion proteins (Figure 3). *E. coli *harboring ABC transporter made a large halo, whereas bacteria lacking the transporter displayed only a small halo (due to leakage of lipase fusion protein from the cells). Fusion proteins with TliA at the C-terminus of GFP, EGF, and CTP were secreted, validating whole TliA as a secretory domain. CTP fused with TliA at the N-terminus did not show the secretory phenotype indicating that the C-terminus of TliA must be free for recognition by ABC transporter. The GFP-TliA fusion protein showed higher lipase activity than the CTP-TliA or EGF-TliA fusion proteins. These observations indicated that GFP might help maintain lipase structure more than EGF and CTP. Consistent with the idea, bacteria harboring the CTP-GFP-TliA fusion protein produced markedly bigger halos than cells containing the CTP-TliA fusion protein. In another experiment, *E. coli *was grown in liquid medium and lipase activity was measured. A similar result to that evident following growth on agar was obtained, with detection of the secretory phenotype of GFP-TliA, EGF-TliA, CTP-TliA, and CTP-GFP-TliA by ABC transporter (Figure 4). Culture supernatant from bacteria harboring CTP-GFP-TliA showed higher lipase activity in liquid medium than the supernatant from bacteria harboring CTP-TliA, reinforcing the role of GFP as a stabilizer. Once again, TliA-CTP was not secreted into the liquid medium because its C-terminus was not free. It was excluded that TliA-CTP had no enzymatic activity because TliA fused at C-terminus were purified as inclusion bodies and refolded to have a lipase activity (data not shown). The results are consistent with the view that the fusion with TliA at the C-terminus of GFP, EGF, and CTP maintained the enzymatic activity of lipase and enabled secretion of fusion proteins.

### Identification of secretion for LARD fusion proteins

**Figure 5:**
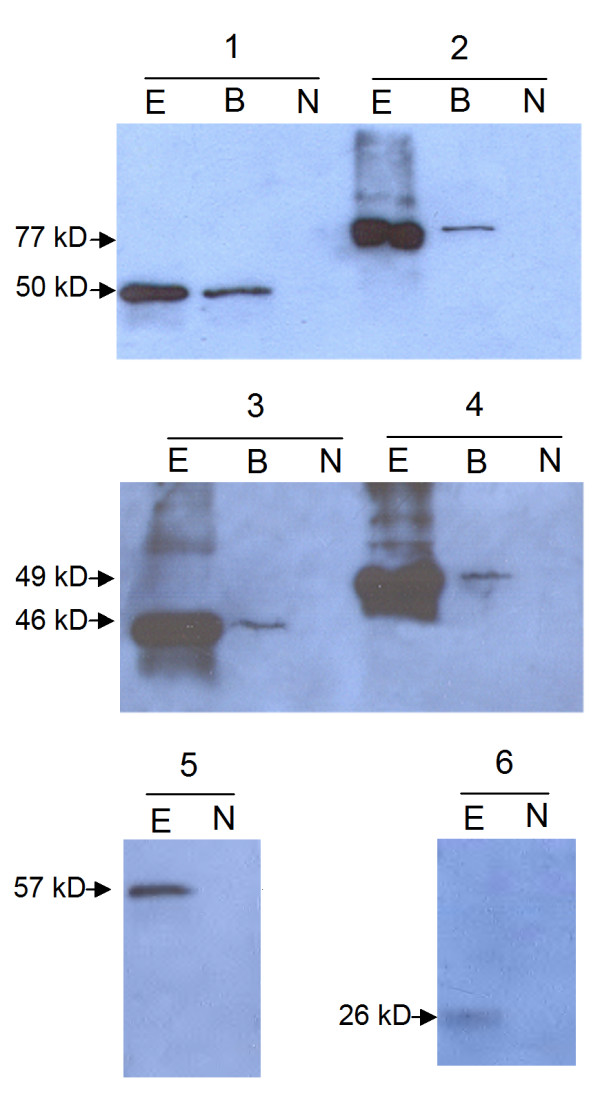
**Immunoblot analysis of recombinant proteins in culture supernatants**. *E. coli *harboring two plasmids was grown for 48 h and 16 μl of the culture supernatant was subjected to SDS-PAGE and analyzed by immunoblot analysis using anti-TliC antibody. E, *E. chrysanthemi *PrtDEF (*E. coli *harboring pEcPrtDEF-184 grown at 37°C); B, *P. fluorescens *TliDEF (*E. coli *harboring pABC-ACYC grown at 25°C); N, no ABC transporter (pACYC-184). 1, pTliA; 2, pGFP-TliA; 3, pGFP-LARD1; 4, pGFP-LARD0; 5, pEGF-TliA; 6, pEGF-LARD1.

While the secretory phenotype of fusion proteins with TliA was evident based on lipase activity, secretion of fusion proteins with LARDs could be detected by Western blotting. Fusion proteins were traced in the culture supernatant using antibody against LARDs. *E. coli *carrying recombinant plasmids containing GFP-LARD and EGF-LARD was cultivated. To precisely identify protein secretion, pEcPrtDEF-184, pABC-ACYC, or pACYC-184 that possessed the PrtDEF transporter, the TliDEF transporter, or which lacked a transporter, respectively, were introduced into *E. coli*, and protein secretion was detected by Western blotting (Figure 5). Although no fusion proteins were secreted in the absence of ABC transporter, GFP-TliA, GFP-LARD0, and GFP-LARD1 fusion proteins were secreted when ABC transporter was present. *E. coli *harboring the PrtDEF of *E. chrysanthemi *secreted more fusion proteins than *E. coli *harboring the TliDEF of *P. fluorescens*. It is likely that the PrtDEF transporter worked better than TliDEF because PrtDEF functioned well at 37°C, the optimum growth temperature for *E. coli*, while TliDEF must be expressed at 25°C for its proper function [12]. In addition, *E. chrysanthemi *ABC transporter PrtDEF might function well in the phylogenetically-neighboring genus *E. coli*, compared to *P. fluorescens *ABC transporter TliDEF. *E. coli *harboring EcPrtDEF secreted fusion proteins including EGF-LARD1 or EGF-TliA. EGF fusion proteins were secreted to the medium, albeit in a reduced amount than GFP fusion proteins. The detected secreted proteins showed the expected molecular weight on Western blotting. There were no detected bands corresponding TliA (49.9 kD), LARD0 (22.2 kD), LARD1 (18.4 kD) or their degraded products in the supernatant of the secreted fusion proteins. When GFP fusion proteins were also detected with antibody against GFP, the same bands detected by the antibody against LARD were detected in the cell and supernatant (data not shown). These results indicated that fusion proteins were contained in the cell and secreted as an intact form of fused proteins. The secreted lipase activities of fusion proteins (Figure 3 and Figure 4) were also confirmed to be derived from the activities of fusion proteins.

## Discussion

There are two distinctive features in the C-terminal signal of proteins secreted by ABC transporter. One is a glycine-rich consensus sequence, GGXGXD, which is repeated many times in the C-terminus. They were firstly found in toxins like HlyA, giving rise to the group name of RTX proteins (repeat in toxins) [29,30]. These repeats constitute high-affinity calcium ion binding sites [23,31]. The secreted proteins can also be distinguished by an extreme C-terminus motif of 4 or 5 residues [32]. The RTX toxins such as HlyA have a preference for hydroxylated residues (Ser and Thr) followed by Ala as a terminal residue [33]. On the other hand, proteases like AprA contain three hydrophobic residues preceded by Asp at the C-terminus [22]. The thermostable lipase, TliA, from which we designed LARD, has four GGXGXD consensus sequences and EGVLIS as its final six C-terminal residues, showing similar organization of several hydrophobic residues preceded by an acidic residue, Glu.

Although C-terminal secretion signals can be identified by the two aforementioned characteristics, precisely how these characteristics participate in secretion is unclear. To understand the interaction between these characteristics and ABC protein, co-crystallization of the C-terminal recognition domain and ABC protein will be required [34]. It is not clear whether primary or secondary/tertiary structures are essential in the recognition of a secretion signal. The RTX secretion signal has been proposed to be largely unstructured [34] based on circular dichroism and nuclear magnetic resonance analyses of isolated RTX secretion signal peptides [35-37]. In addition, it has been suggested there is no common primary or secondary structure except RTXs and the extreme C-terminal motif [34], implying that there is no need of secondary structure for the binding to ABC transporter. However, three proteases secreted by ABC transporter, of which structures are elucidated, in *P. aeruginosa *[23], *Serratia marcescens *[24] and *E. chrysanthemi *[25] have a β-roll C-terminal structure. Studies on isolated β-roll domain from RTX toxins were performed in *Bordetella pertussis *adenylate cyclase toxin [38] and *E. coli *hemolysin [39]. RTX with eight GGXGXDXLXs that was chemically synthesized was shown to form β-roll structure [40]. From these previous reports, we thought that the whole C-terminal β-roll domain was needed for secretion and chaperon/folding. We designed two LARDs on the basis of the predicted C-terminal structure of lipase, which is derived from the structure of *P. aeruginosa *alkaline protease, because the structure of *P. fluorescens *SIK W1 lipase had remained unresolved. Recently, two similar lipase structures were resolved and shown to have the same β-roll structure in their C-terminal region [41,42]. They are somewhat different from TliA in that they have 13 copies of glycine-rich consensus sequence and a pair of β-rolls (β-roll sandwich) in contrast to the four glycine-rich consensus sequences and one β-rolls that is in TliA.

Presently, we could assess the importance of the extreme C-terminus motif in the secretion of CTP fusion proteins. The CTP peptide (11 aa) was originally designed not for the secretion of itself, but for further application in introduction of fused proteins into eukaryotic cytoplasm. It was too short to assess the secretion of LARDs and the tendency to be produced as inclusion body in *E. coli *[21] made it unqualified for a secreted model protein. The CTP was fused with whole lipase and the secretory phenotype of CTP fusion was observed on the solid medium (Figure 3). CTP-lipase showed the secretory phenotype by supplementing the ABC transporter but lipase-CTP did not, consistent with a previous report in which the necessity for COOH terminal exposure of the extreme C-terminus motif was demonstrated; addition of even one amino acid impairs the secretion [32]. In another study, the elimination of Glu in ELLAA in the extreme C-terminus of *S. marcescens *LipB and re-positioning in all the possible positions in the downstream sequence had no detectable effect upon the secretion of the lipase, implying that the extreme C-terminal motif is not essential for the secretion [43]. The latter authors proposed that a new region near extreme C-terminal motif is necessary for recognition by the ABC transporter. These observations highlight the ongoing uncertainty as to which part of C-terminal signal is recognized by the ABC transporter. It is unclear whether the rigid β-roll structure is required or not for secretion through the ABC transporter, despite the popularity of the theory that C-terminal signals are unstructured during a process of secretion but needed for chaperon/folding is prevailing [34,38,40]. Research is ongoing to check the possibility of shorter LARDs, through decreased LARD sizes from LARD1 (residues 303–476 of TliA) to LARD5 (residues 443–476).

Previously, Palacios et al. attached 181-residue C-terminal signal sequence to eukaryotic proteins such as endochitinase, GFP, hEPO (human erythropoetin) and tGH (trout growth hormone) [44]. The endochitinase and GFP were exported by *E. chrysanthemi *PrtDEF but hEPO and tGH were not. They reasoned that the failure of exporting hEPO and tGH was derived from the disulfide bond formation inside the cell. We used GFP and EGF as model proteins in the export experiments. Although GFP has no disulfide bridge, EGF has three disulfide bridges. In our experiment, EGF was exported in *E. coli*, showing the possibility that the protein having disulfide bonds can be exported. More thorough experiments are needed to elucidate the relationship between disulfide bond bridge and protein export.

## Conclusion

We designed LARDs based on comparative modeling of *P. fluorescens *lipase and attached them genetically to GFP and EGF. Two different LARDs (LARD0 and LARD1) contained β-roll structure and either Pro-Gly linker or Factor Xa site between fusion proteins and LARDs. We also attached the whole lipase (TliA) genetically to GFP, EGF and CTP. The fused proteins with the whole lipase were secreted in *E. coli *with the ABC transporter and showed lipase activity as an intact fused form in the supernatnat. The GFP and EGF fused with LARDs or TliA were exported into the extracellular medium in *E. coli *containing ABC transporter of *P. fluorescens *and *E. chrysanthemi*. In this report, only *E. coli *was explored as an expression host for the possibility of ABC transporter for recombinant protein production. The secretory expression of fusion proteins in *E. coli *will extend to those in *P. fluorescens *in which the ABC transporter TliDEF are better expressed [13]. *P. fluorescens *supplemented with TliDEF produced extracellular lipase up to about 15% total proteins [13]. An efficient protein manufacturing factory is expected to be constructed using *Pseudomonas *as a host.

## Methods

### Bacterial strains, plasmids and DNA manipulation

*E. coli *XL1-Blue was used as a host strain for DNA manipulation and gene expression. Plasmids pCTP [21], pKK223-3 (Amersham Pharmacia, Piscataway, NJ), and pBluescript-SK(+) (Stratagene, San Diego, CA) were used as vectors. All DNA manipulations including restriction endonuclease digestion, ligation, transformation, and agarose gel electrophoresis were carried out by standard procedures [45]. All restriction enzymes, DNA-modifying enzymes, and related reagents used for DNA manipulation were purchased from Takara Shuzo (Shiga, Japan), Solgent (Daejeon, Korea) or Sigma-Aldrich (St. Louis, MO).

### Plasmid construction

Table 1 shows plasmids used in this study and Table 2 shows polymerase chain reaction (PCR) products used in plasmid construction. Two or three different PCR-amplified DNAs were inserted into multicloning site of plasmids in frame to make a continuous fusion protein (Table 1). GFP coding sequence without stop codon was obtained by PCR amplification using pGFPuv (Clontech, Mountain View, CA) as a template (Table 2). TliA, LARD0 and LARD1 were also PCR-amplified using pTOTAL [12] as a template and primers containing different enzyme sites. A repetitive Pro-Gly linker was added at the N-terminus of LARD0 using the PCR primer used for the amplification of LARD0. Factor Xa site was also added at N-terminus of LARD1 using the PCR primer. EGF was PCR-amplified using EGF-containing plasmid pGEMT-hEGF and EGF-fusion proteins were constructed similarly as GFP-fusion proteins. The CTP fusion was constructed by inserting TliA or LARDs into pCTP [21].

**Table 1:** Characteristics of plasmids used in this study.

**Plasmids**	**Properties/inserts**	**Vector/Source**
pGFPuv	Cycle 3 variant of GFP	pUC/Clontech
pGFP-TliA	GFP insert 1, TliA insert1	pKK223-3 [48]
pGFP-SXold	intermediate to pGFP-LARD0	pBluescript-SK(+)
pGFP-LARD0	GFP insert 1, LARD0 insert	pBluescript-SK(+)
pGFP-LARD1	GFP insert 2, LARD1 insert	pKK223-3
pEGF-TliA	EGF insert, TliA insert1	pKK223-3
pEGF-LARD1	EGF insert, LARD1 insert	pKK223-3
pCTP-TliA	TliA insert 2	pCTP [21]
pTliA-CTP	TliA insert 3	pCTP
pCTP-GFP-TliA*	GFP-TliA insert	pCTP
pCTP-GFP-LARD0	GFP-LARD0 insert	pCTP
pCTP-GFP-LARD1	GFP-LARD1 insert	pCTP
pABC-ACYC	*tliDEF *from *P. fluorescens*	[12]
pEcPrtDEF-184	*prtDEF *from *E. chrysanthemi*	pRUW4 [49]

The name of plasmids indicates the order of different genes. For example, pCTP-GFP-TliA* denotes a fusion protein consisting of CTP, GFP, and TliA in order.

**Table 2:** Oligonucleotide primers used for DNA inserts in plasmid construction.

**Insert**	**Primer sequence**	**Restriction sites/feature**
GFP insert 1	GGG GAATTC ATGAGTAAAGGAGAAGAACTTT	EcoRI
	GGG TCTAGA*GGCGGCGGC *TTTGTAGAGCTCATCCATGCC	XbaI, AAA
GFP insert 2	GCG CCGCGG TG ATGAGTAAAGGAGAAGAACTTT	SacII
	GGG TCTAGA CC *TGGACCACCACC*TTTGTATAGTTCATCCATGCCA	XbaI, GGGP
LARD0 insert	GCG ATCGAT A *(CCAGGT) × 5 *TCGTCCCTCGGCGTGCATG	ClaI, PGPGPGPGPG
	GCG GGTACC TCAACTGATCAGCACACCCT	KpnI
LARD-1 insert	AC TCTAGA*ATTGAAGGACGA *TCCATCGCCAACCTGTCG	XbaI, F.Xa
	GGG AAGCTT ATGAACCGCCGATAATCCGT	HindIII
TliA insert 1	AC TCTAGA*ATTGAAGGACGA *ATGGGTGTATTTGACTACAAGA	XbaI, F.Xa
	GGG AAGCTT ATGAACCGCCGATAATCCGT	HindIII
TliA insert 2	GGG GGTACC*GGAGGA *ATGGTGATTTGACTACAAGA	KpnI, GG
	GGG AAGCTT TCAACTGATCAGCACACCCT	HindIII
TliA insert 3	GGG ATCGAT*GGA *ATGGGTGTATTTGACTACAAGA	ClaI, G
	GGG GGATCC TCCTCC ACTGATCAGCACACCCTCG	BamHI
EGF insert	GGG GAATTC ATGAATAGTGACTCTGAATGTCC	EcoRI
	GGG TCTAGA GCGCAGTTCCCACCACTTC	XbaI
GFP-LARD0 insert	GCG GGTACC*CCAGGTGGT *ATGACCATGATTACGCCAAG	KpnI PGPG
	TAATACGACTCACTATAGGG	(KpnI)
GFP-TliA insert	CG GGTACC*CCAGGT *ATGAGTAAAGGAGAAGAACTTT	KpnI, PG
GFP-LARD1 insert	ATCTTCTCTCATCCGCCAAA	(HindIII)

A: alanine, P: proline, G: glycine, F.Xa: Factor Xa site

Enzyme sites are underlined and feature sequences are italicized.

(enzyme) denotes an enzyme site upstream of reverse primer.

### LARD design

*P. aeruginosa *alkaline protease (AprA) has a N-terminal activity domain and C-terminal β-roll (or β-sandwich) structure (PDB code: 1kap). The C-terminal part of AprA has a low similarity with TliA (20% identity). This β-roll structure is thought to be the secretion signal for the ABC transporter system. We built the C-terminal structure of TliA using the SWISS-MODEL program [46] and the structure of alkaline protease, and the similar β-roll structure was built by homology modeling. The LARD was designed in two ways. Amino acid residues 269 to 476 (SSLG-VLIS) and 303 to 476 (SIAN-VLIS) of TliA were defined as LARD0 and LARD1, respectively.

### Growth conditions

Luria-Bertani (LB) medium supplemented with ampicillin (50 μg/ml) and chloramphenicol (34 μg/ml) was used for cultivating recombinant *E. coli *XL1-Blue. Each *E. coli *cultures was generated in a 12 ml culturing tube containing 2 ml of LB medium in a shaking incubator at 180 rpm, reaching OD_600 _2 to 2.5. The culture temperature was selected as 25°C for cultures harboring pABC-ACYC (tliDEF) and 37°C for cultures harboring pEcPrtDEF-184 (prtDEF) because each ABC transporter system functions optimally at those temperatures. Cultures harboring pACYC-184 (no ABC transporter) was grown at 37°C because *E. coli *grows best at this temperature. Each culture was induced with 0.05 mM isopropyl-beta-D-thiogalactopyranoside (IPTG), where the secretion efficiency of TliA with the TliDEF transporter system is highest in *E. coli*.

### Antibodies

Polyclonal antibodies against the C-terminal signal sequence (TliC) were produced with a synthetic peptide (YQPTDRLVFQGADGST, residues 421–436 of TliA). The peptide was coupled to the immunogenic carrier protein keyhole limpet hemocyanin (KLH) via an additional N-terminal cysteine of each peptide by N-γ-maleimidobutyryloxylsuccinimide (GMBS) conjugation [47]. Immunization of each peptide and sampling of anti-serum from rabbits were performed by a commercial facility (Peptron, Daejeon, Korea). Antibody was purified by the peptide-linked affinity resin, which was prepared by linking peptide to the activated affinity resin. Crude serum was applied to the affinity column, and anti-TliC immunoglobulin G (IgG) was eluted with 100 mM glycine (pH 2.5), neutralized by 1 M Tris-HCl (pH 8.0), and dialyzed in phosphate buffered saline (PBS).

### Immunoblot analysis of secreted protein

For detection of the secretion of TliA or LARD fusion proteins expressed by the cultivation of *E. coli *XL1-Blue harboring recombinant plasmids, 12 μl culture supernatants of recombinant *E. coli *cells were subjected to sodium dodecyl sulfate-polyacrylamide gel electrophoresis (SDS-PAGE) and then were electro-transferred onto a polyvinylidene fluoride (PVDF) membrane (Amersham). Proteins were detected by immunoblotting with anti-TliC serum, followed by binding of peroxidase-conjugated anti-rabbit IgG, and signals were detected with the enhanced chemiluminescence system (Amersham).

### Lipase activity

To identify a secretion phenotype on solid medium, *E. coli *was grown at 25°C for 48 h on LAT (LB medium, 1.5% Bacto Agar, 0.5% tributylin). The phenotype was evident by the development of a halo due to the secreted lipase [12]. In addition, lipase activity was measured spectrophotometrically using p-nitrophenyl palmitate (pNPP) as a substrate [12]. Ten millimolar pNPP dissolved in acetonitrile was mixed with ethanol and 50 mM Tris-HCl (pH 8.5) to a final ratio of acetonitrile:ethanol:Tris-HCl of 1:4:95 (v/v/v). The reaction was started by adding 50 μl of culture supernatant to 200 μl of reaction mixture at 42°C, and absorbance at 420 nm was monitored with a Magellan microplate reader (Tecan, Männedorf, Switzerland) for 20 min. The activity was measured by the increase of optical density (OD).

## Abbreviations

TliA: thermostable lipase A; ABC: ATP binding cassette; LARD: lipase ABC transporter domains; GFP: green fluorescent protein; EGF: epidermal growth factor; CTP: cytoplasmic transduction peptide; AprA: alkaline protease A; TliDEF: ABC transporter for TliA; PrtDEF: ABC transporter for PrtA; RTX: repeat in toxins.

## Competing interests

The authors declare that they have no competing interests.

## Authors' contributions

CWC and played leading role in all experiments and drafted the manuscript. JY and KK participated all experiments. YM contributed in experimental design and helped writing manuscript. HK performed a comparative modeling of TliA. JHA designed experiments and interpreted results. All authors read and approved the final manuscript.

## Authors' information

CWC, JY and KK participated this research project as secondary school students (11^th ^grade) and showed their concentration and brilliance.
